# The relationship of socio-demographic characteristics and knowledge of breast cancer on stage of behavioral adoption of breast self-examination

**DOI:** 10.3934/publichealth.2020049

**Published:** 2020-08-06

**Authors:** Soo-Foon Moey, Aaina Mardhiah Abdul Mutalib, Norfariha Che Mohamed, Nursyahirah Saidin

**Affiliations:** Department of Diagnostic Imaging & Radiotherapy, Kulliyyah of Allied Health Sciences, International Islamic University Malaysia (IIUM), Kuantan Campus, Pahang, Malaysia

**Keywords:** breast self-examination, behavioral adoption, breast cancer, socio-demographic, knowledge

## Abstract

**Background/aim:**

In Malaysia, breast cancer is the most common cancer among women. As such, early diagnosis and screening practices are important to increase the survival rate. Breast self-examination (BSE) is one of the main screening methods for breast cancer. Socio-demographic characteristics and knowledge of breast cancer are amongst the crucial roles in determining women's behavioral adoption in performing BSE. This study aims to assess the relationship of socio-demographic factors and knowledge of breast cancer on the stage of behavioral adoption of BSE among Malaysian women in Kuantan, Pahang.

**Materials and methods:**

A cross-sectional study was conducted on 520 women from three different government health clinics in Kuantan and IIUM Family Health Clinic from February to April 2018. Data were collected using a self-administered questionnaire on socio-demographic factors and knowledge of breast cancer and its effect on the behavioral adoption of BSE.

**Results:**

Significant difference was found between socio-demographic characteristics and behavioral adoption of BSE. However, only breast screening and the best time for screening were found to be significant with the behavioral adoption of BSE and knowledge of breast cancer.

**Conclusion:**

It is found that most women in Kuantan, Pahang perform BSE but were still unaware of the importance of performing BSE for early breast cancer detection. This study was expected to enhance women's awareness of the benefits of performing BSE.

## 1. Introduction

Breast cancer is the most frequent cause of death among women globally [1]. In Malaysia, the age-standardized incidence (ASR) rate for breast cancer is 38.46 per 100,000 women [2] indicating that one out of every 19 Malaysian women has the chance of getting breast cancer during their lifetime [3]. More than half of the new cases of breast cancer in Malaysia were diagnosed in women below 50 years of age [4] at an advanced and metastatic stage [5]. This indicates that with early detection, the 5-year survival rate is 92% or higher. As such, the importance of early breast cancer screening [6].

Mammography, clinical breast examination (CBE) and breast self-examination (BSE) are effective methods for early breast cancer detection [7]. As mammography is costly and may not be within the access of those with lower socioeconomic status [1], BSE is useful as a screening tool more so in less developed countries in detecting the presence of abnormalities in the breast [8] as it creates an opportunity for women to detect any changes in the breasts. Thus, with the right technique in performing BSE, it will contribute to early diagnosis and treatment options [9]. Even though there are potential false-negative findings in BSE due to the incompetence of women in performing it [8], BSE is recommended because it does not involve any cost, can be carried out by the woman herself, painless and does not require any specific equipment [10].

Knowledge of breast cancer is important as it will encourage women to perform breast cancer screening [11] as knowledge of breast cancer leads to an improvement of breast cancer interventions [12]. This is because a study found that inadequate knowledge prevents women from seeking treatment, thus contributing to high mortality [13]. As such, early detection would result in a better prognosis and treatment of the disease [14].

Unfortunately, the behavioral adoption of BSE is prevalently low amongst Asian women [12],[15] and is characterized mainly by women's psychosocial attributes and socio-demographic characteristics [16] probably due to ignorance of its' benefits [17]. As such, the Trans-theoretical model (TTM) was utilized in determining the stage of behavioral adoption of BSE amongst study respondents to help them to be at better stages with suitable interventions [18].

**Figure 1.**
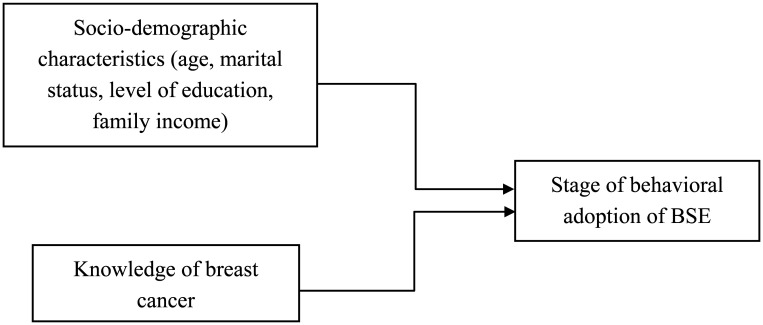
Hypothetical conceptual framework of the study.

It appears that education, income, age and marital status are related to BSE conformance [16] as previous studies indicated that most women with lower socio-economic status (education and occupation) in Asia remain unaware of breast cancer and the correct techniques of performing BSE [19]. Further, previous similar studies were conducted mainly in developed Asian countries like Singapore [20] and South Korea [21]. In Malaysia, the study on the knowledge of breast cancer is usually conducted amongst university students [2], working women [22] and those living in urban centers [6]. As such, the objectives of this study is to determine the association between knowledge of breast cancer and socio-demographic characteristics on the stage of behavioral adoption of BSE amongst women in Kuantan, Pahang. The hypothetical conceptual framework for the study is as in Figure 1.

## 2. Materials and method

### 2.1. Study design

This cross-sectional study was used to study the relationship between knowledge of breast cancer and socio-demographic characteristics on the stage of behavioral adoption of BSE amongst women in Kuantan, Pahang.

### 2.2. Sample size and setting

The study was conducted from February to April 2018 amongst 520 Malaysian women living in Kuantan, Pahang aged 35 to 70 years old. A multistage, cluster-stratified random sampling method was carried out to obtain the appropriate sample size. In the first stage, a cluster sampling method was implemented by randomly selecting three sub-districts in Kuantan, Pahang. Following that, a stratified sampling method was carried out to randomly select the polyclinic in each sub-district. Thus, Klinik Kesihatan Beserah from Beserah and Klinik Kesihatan Balok from Sungai Karang were selected. However, two polyclinics; Klinik Kesihatan Kuantan and IIUM Family Health Clinic were randomly selected from Kuala Kuantan since the region was larger and had more residents compared to Beserah and Sungai Karang. The sample size was calculated using a single proportion formula based on the assumption of 5% type 1 error *p* < 0.05, a precision/absolute error at 2%. Based on the calculation, a sample size of 520 respondents was required and criteria of the respondents are as in Appendix A were randomly selected to participate in the study.

### 2.3. Data collection procedure

The respondents were briefed about the study beforehand and willingness to fill the questionnaire was considered as consent to participate in the study. The respondents were also notified that their participation in the study was voluntary and they can withdraw from the study at any time if they do not want to be a part of the study.

Ethical principles were followed throughout the study and ethical approval was acquired from the Kulliyyah Postgraduate Research Committee (KPGRC) (approval number: KAHS 173), followed by IIUM Research Ethics Committee (IREC) (approval number: 2017-075) and Medical Research and Ethics Committee (MREC) (approval number: NMRR-17-2131-37586 (IIR).

### 2.4. Instruments

The questionnaire was constructed based on a review from previous research literature of BSE, stage of behavioral adoption of BSE and knowledge of BSE. Five health professional experts including two professors, a radiologist specializing in diagnosis and screening of breast cancer, an English lecturer and a research scholar in women's health were involved in validating the content of the questionnaire. The self-administered questionnaire comprised of three sections. Section one covers socio-demographic characteristics (age, marital status, level of education and family income). Section two comprised of 26 questions that measured respondents' knowledge of breast cancer. It consisted of seven questions on the symptoms of breast cancer, seven questions on the risk factors of breast cancer, seven questions on the method of breast screening, three questions on the best time for breast screening and two questions on the perceptions of breast lump. A dichotomous type questionnaire was used in this section to elicit the respondent's knowledge of breast cancer. Each question answered correctly was given a score of 1 and the question answered incorrectly or no answer was given a score of 0. Section three consists of questions pertaining to the stage of behavioral adoption of BSE (Appendix B).

### 2.5. Pilot study

Prior to the full-scale research, a pilot study was carried out on 103 respondents who were randomly selected. The steps taken in the pilot study included content validation and translation to maintain and ensure the overall accuracy of the questionnaire. Additionally, exploratory factor analysis (EFA) was used to explore the construct validity of the questionnaire. Kaiser-Meyer-Olkin (KMO) and Bartlett's Test of Sphericity was also used to measure the adequacy of each item in the questionnaire. During several steps of EFA, few factors were fix and problematic items were eliminated one by one as they failed to meet the minimum criteria of having factor loading ≥ 0.40 or cross-loading on other factors. Findings from the EFA revealed nine factors that jointly accounted for 74.2% of the observed variance. All nine factors have good internal consistency with Cronbach's alpha ≥ 0.8. The questionnaire also showed good convergent and discriminant validity [23].

### 2.6. Statistical analysis

All data were analyzed using SPSS version 21.0 (IBM Corporation, Armonk, NY, USA). Descriptive statistics and chi-square were used to assess the relationship between the stage of behavioral adoption of BSE and socio-demographic variables. Multinomial logistic regression was performed to examine the relationship between the stage of behavioral adoption of BSE and knowledge of breast cancer.

## 3. Results

### 3.1. Socio-demographic characteristics of the respondents

The socio-demographic characteristics of the respondents are as in Table 1. The average age of the respondents was 44.64 years old (SD = 9.513). The majority of the respondents were in the age group of 35 to 40 years (46.5%), married (79.6%) and obtained tertiary education (52.5%) with a family income of between RM 3000 to RM 5999 (37.9%). It was found that the largest number of women who performed BSE were between 35 to 40 years old (46.5%) and the least was between 66 to 70 years old (2.1%).

### 3.2. Knowledge of breast cancer

There were 26 questions on knowledge of breast cancer and most of the respondents were able to answer more than 13 questions correctly (59.0%). From Table 2, the results show that most of the respondents understand the symptoms of breast cancer, risk factors of breast cancer, methods of breast screening, best time for breast screening and perception of breast lump. As for symptoms of breast cancer, most of the respondents indicated that lump in the breast (74.0%), bloody discharge from the nipple (62.7%), puckering of the skin of the breast (52.3%), swelling of the axillary lymph node (62.1%) and warmth and redness throughout the breast (60.6%) as the correct symptoms for symptoms of breast cancer. However, only 49.6% and 25.6% of the respondents respectively, indicated nipple retraction and weight gain after menopause were symptoms for breast cancer. For risk factors of breast cancer, most of the respondents indicated that a diet high in fats (55.0%) and previous history of breast cancer (74.8%) are risk factors of breast cancer. However, the respondents know that onset of menses before 12 years of age (32.7%), menopause after 55 years old (38.8%), first pregnancy after 35 years old (37.7%), nulliparity at age 40 years (41.2%) and taking oral contraceptive pills (OCP) (41.3%) are also the risk factors of breast cancer.

Most of the respondents indicated that BSE (91.7%), CBE (85.4%), mammography (74.8%), ultrasound (55.4%) and MRI (52.1%) are some of the breast screening methods. Additionally, 55.2% of the respondents indicated that a Pap smear test was not a method of breast cancer screening. The respondents were asked on the best time of breast screening in which the correct answer was a week after menstruation and most of them (54.6%) answered correctly. Most of the respondents answered wrongly for presence of an abnormal lump in the breast means cancer (45.4%) and pain in the breast lump is cancer (58.8%).

**Table 1.** Socio-demographic characteristics of respondents (n = 520).

		Frequency	Percent (%)	Cumulative Percent (%)
Age
	35–40	242	46.5	46.5
	41–45	80	15.4	61.9
	46–50	66	12.7	74.6
	51–55	55	10.6	85.2
	56–60	46	8.8	94.0
	61–65	20	3.8	97.9
	66–70	11	2.1	100.0
Marital status
	Single	69	13.3	13.3
	Married	414	79.6	92.9
	Divorcee	13	2.5	95.4
	Widow	24	4.6	100.0
Level of education
	No formal education	2	0.4	0.4
	Primary education	31	6.0	6.3
	Secondary education	214	41.2	47.5
	Tertiary education	273	52.5	100.0
Family income (RM)
	<1000	60	11.5	11.5
	1000–2999	173	33.3	44.8
	3000–5999	197	37.9	82.7
	6000–9999	56	10.8	93.5
	>10000	34	6.5	100.0

RM: Ringgit Malaysia.

**Table 2.** Knowledge of breast cancer (n = 520).

Item		% Correct
Symptoms of breast cancer	Lump(s) in the breast	74.0
	Nipple retraction (drawn inward)	49.6
	Bloody discharge from the nipple (bloody fluid seeps out from nipple)	62.7
	Puckering (dimpling) of the skin of the breast	52.3
	Swelling of the axillary's lymph	62.1
	Warmth (burning) and redness throughout the breast	60.6
	Weight gain after menopause	25.6
Risk factors of breast cancer	Onset menses before 12 years old	32.7
	Menopause after 55 years old	38.8
	Diets high in fats	55.0
	Past history of breast cancer	74.8
	First pregnancy after 35 years old	37.7
	Nulliparity at age 40 years (Women who have no children or who their first child after the age 40 years)	41.2
	Taking oral contraceptive pills (OCP)	41.3
Method of breast screening	Breast Self-Examination	91.7
	Clinical Breast Examination	85.4
	Pap smear test*	55.2
	Mammography	74.8
	Genetic screening test	47.3
	Ultrasound	55.4
	Magnetic Resonance Imaging (MRI)	52.1
Best time for screening	Before or during menstrual periods*	58.5
	After one week of menstrual periods	54.6
	Same date every month*	60.6
Perception on breast lump	Presence of abnormal lump(s) in the breast means cancer*	45.4
	Pain of the breast lump means cancer (mastodynia)*	58.8

*: false answer.

### 3.3. Stages of behavioral adoption of BSE

Table 3 shows the stage of behavioral adoption of BSE amongst respondents. Most of the respondents were at the action stage 172 (33.1%) followed by the contemplation stage 106 (20.4%) and the least number of respondents were at the relapse stage 13 (2.5%).

### 3.4. The relationship between socio-demographic characteristics and stage of behavioral adoption of BSE

A Chi-square table was carried out to determine the relationship between socio-demographic characteristics and stage of behavioral adoption of BSE (Table 4). A significant relationship was found between socio-demographic characteristics and the stage of behavioral adoption of BSE.

**Table 3.** The stages of behavioral adoption of BSE amongst respondents.

Stage of behavioral adoption of BSE	No of respondents (n = 520)	Percentages (%)
Pre-contemplation	57	10.9
Contemplation	106	20.4
Preparation	69	13.3
Action	172	33.1
Maintenance	103	19.8
Relapse	13	2.5

**Table 4.** Association between socio-demographic characteristics and stage of behavioral adoption of BSE.

Variable		Stage of behavioral adoption of BSE							x^2^
		N	1	2	3	4	5	6	(df),
									*p*-value
Age	35–40	242 (46.5)	31 (6.0)	64 (12.3)	34 (6.5)	77 (14.8)	35 (6.7)	1 (0.2)	(124.546)
									30,
									*p* < 0.01
	41–45	80 (15.3)	8 (1.5)	19 (3.7)	13 (2.5)	19 (3.7)	17 (3.3)	4 (0.8)	
	46–50	66 (12.6)	2 (0.4)	11 (2.1)	8 (1.5)	30 (5.8)	14 (2.7)	1 (0.2)	
	51–55	55 (10.5)	2 (0.4)	8 (1.5)	9 (1.7)	18 (3.5)	16 (3.1)	2 (0.4)	
	56–60	46 (8.8)	6 (1.2)	4 (0.8)	3 (0.6)	22 (4.2)	10 (1.9)	1 (0.2)	
	61–65	20 (3.8)	3 (0.6)	0 (0.0)	2 (0.4)	5 (1.0)	10 (1.9)	0 (0.0)	
	66–70	11 (2.1)	5 (1.0)	0 (0.0)	0 (0.0)	1 (0.2)	1 (0.2)	4 (0.8)	
Marital status	Single	69 (13.2)	11 (21)	32 (6.2)	9 (1.7)	8 (1.5)	9 (1.7)	0 (0.0)	(72.167)
									15,
									*p* < 0.01
	Married	414 (79.6)	37 (7.1)	73 (14.0)	58 (11.2)	152 (29.2)	85 (16.3)	9 (1.7)	
	Divorcee	13 (2.5)	2 (0.4)	1 (0.2)	2 (0.4)	3 (0.6)	4 (0.8)	1 (0.2)	
	Widow	24 (4.6)	7 (1.3)	0 (0.0)	0 (0.0)	9 (1.7)	5 (1.0)	3 (0.6)	
Level of education	No formal education	2 (0.3)	1 (0.2)	1 (0.2)	0 (0.0)	0 (0.0)	0 (0.0)	0 (0.0)	(79.628)
									15,
									*p* < 0.01
	Primary education	31 (5.9)	11 (2.1)	2 (0.4)	2 (0.4)	5 (1.0)	9 (1.7)	2 (0.4)	
	Secondary education	214 (41.1)	18 (3.5)	22 (4.2)	23 (4.4)	95 (18.3)	46 (8.8)	10 (1.9)	
	Tertiary education	273 (52.5)	27 (5.2)	81 (15.6)	44 (8.5)	72 (13.8)	48 (9.2)	1 (0.2)	
Family income (RM)	<1000	60 (11.9)	11 (2.1)	5 (1.0)	12 (2.3)	13 (2.5)	14 (2.7)	5 (1.0)	(46.945)
									20,
									*p* < 0.01
	1000–2999	173 (33.2)	25 (4.8)	32 (6.2)	17 (3.3)	63 (12.1)	30 (5.8)	6 (1.2)	
	3000–5999	197 (37.8)	12 (2.3)	45 (8.7)	25 (4.8)	72 (13.8)	41 (7.9)	2 (0.4)	
	6000–9999	56 (10.7)	9 (1.7)	12 (2.3)	10 (1.9)	13 (2.5)	12 (2.3)	0 (0.0)	
	>10000	34 (6.5)	0 (0.0)	12 (2.3)	5 (1.0)	11 (2.1)	6 (1.2)	0 (0.0)	

N: Number; RM: Ringgit Malaysia; 1: pre-contemplation; 2: contemplation; 3: determination/preparation; 4: action; 5: maintenance; 6: relapse.

### 3.5. The relationship between constructs of knowledge of breast cancer and stage of behavioral adoption of BSE

Multinomial logistic regression was used to determine the relationship between constructs of knowledge on breast cancer and stage of behavioral adoption of BSE. The outcome occurrence likelihood was determined using the odds ratio (OR) at a 95% confidence interval. From the model fitting information in Table 5, the final model was better than the intercept model (*p* < 0.05). With reference to the relapse stage, the method of breast screening was found to be significant with the pre-contemplation stage (OR: 0.545, CI: 0.362–0.822, *p*: 0.004) and the determination stage (OR: 0.207, CI: 0.444–0.998, *p*: 0.666). Further, the best time of screening was found to be significant with contemplation (OR: 1.572, CI: 1.032–2.395, *p*: 0.035), determination (OR: 1.845, CI: 1.199–2.839, *p*: 0.005), action (OR: 1.767, CI: 1.167–2.676, *p*: 0.007) and maintenance (OR: 1.568, CI: 1.030–2.388, *p*: 0.036). No significant differences were indicated between the risk of breast cancer, symptoms of breast cancer and perception of breast lump with the stage of behavioral adoption of BSE. Table 6 shows the multivariate relationship between knowledge on breast cancer and stage of behavioral adoption of BSE amongst respondents.

**Table 5.** Model fitting criteria on the relationship of knowledge constructs and stage of behavioral adoption of BSE.

Model	Model Fitting Criteria	Likelihood Ratio Tests		
	−2 Log Likelihood	Chi-Square	Df	*p*-value
Intercept Only	1589.210			
Final	1524.958	64.252	25	*p* < 0.05

**Table 6.** Multivariate relationship between knowledge on breast cancer and the stage of behavioral adoption of BSE amongst respondents.

Stage of behavioral adoption of BSE		B	Std. Error	*p-*value	Exp (B)	95% CI for Exp (B)	
						Lower Bound	Upper Bound
Pre-contemplation	Risk of breast cancer	0.261	0.362	0.471	1.299	0.638	2.642
	Best time for screening	0.422	0.225	0.061	1.526	0.981	2.373
	Symptoms of breast cancer	−0.037	0.166	0.826	0.964	0.696	1.335
	Perception of breast lump	−0.030	0.373	0.936	0.970	0.467	2.017
	Method of breast screening	−0.606	0.209	0.004	0.545	0.362	0.822
Contemplation	Risk of breast cancer	0.324	0.348	0.351	1.383	0.699	2.736
	Best time for screening	0.453	0.215	0.035	1.572	1.032	2.395
	Symptoms of breast cancer	0.097	0.157	0.539	1.102	0.809	1.500
	Perception of breast lump	0.081	0.357	0.821	1.084	0.539	2.181
	Method of breast screening	−0.501	0.201	0.013	0.606	0.409	0.898
Determination	Risk of breast cancer	0.464	0.359	0.196	1.591	0.787	3.214
	Best time for screening	0.612	0.220	0.005	1.845	1.199	2.839
	Symptoms of breast cancer	−0.065	0.163	0.689	0.937	0.680	1.290
	Perception of breast lump	0.109	0.368	0.766	1.116	0.542	2.297
	Method of breast screening	−0.407	0.207	0.049	0.666	0.444	0.998
Action	Risk of breast cancer	0.220	0.342	0.520	1.246	0.637	2.437
	Best time for screening	0.569	0.212	0.007	1.767	1.167	2.676
	Symptoms of breast cancer	−0.101	0.153	0.511	0.904	0.669	1.221
	Perception of breast lump	0.077	0.350	0.826	1.080	0.544	2.144
	Method of breast screening	−0.257	0.196	0.190	0.773	0.526	1.136
Maintenance	Risk of breast cancer	0.398	0.349	0.255	1.488	0.751	2.951
	Best time for screening	0.450	0.214	0.036	1.568	1.030	2.388
	Symptoms of breast cancer	0.012	0.157	0.941	1.012	0.744	1.376
	Perception of breast lump	0.325	0.358	0.364	1.384	0.686	2.791
	Method of breast screening	−0.226	0.202	0.263	0.798	0.537	1.184

Exp (B): Odds ratio; CI: Confidence interval.

## 4. Discussion

This study is to determine the relationship between socio-demographic characteristics (age, marital status, level of education and family income) and knowledge of breast cancer (risk of breast cancer, the best time for screening, symptoms of breast cancer, the perception of breast lump and method of breast screening) with the stage of behavioral adoption of BSE.

### 4.1. Socio-demographic characteristics on stage of behavioral adoption of BSE

A statistical significance was found between constructs of socio-demographic characteristics and stage of behavioral adoption of BSE. This relates to previous studies whereby an increase in age will reduce the performance of BSE [24]. It was found that women aged less than 50 years held stronger positive attitudes towards performing BSE than the older age group. This is because they believe that performing BSE can detect breast cancer at an early stage [25]. Older women, on the other hand probably refused to perform BSE due to a lack of confidence and ignorance on breast cancer knowledge [24]. Additionally, past studies also showed that older women did not like to touch their bodies and felt embarrassed to do so [26]. This probably arises as they perceived that they are not susceptible to breast cancer [27]. Marital status also showed a significant association with the stage of behavioral adoption of BSE with most of the respondents being married women. In accordance with previous studies, it was suggested that married women were more conscious of their breasts and were more likely to perform BSE [28]. This is because a married woman has the advantage of having a household, economic and emotional support from their spouse [29],[30] as spousal emotional and economic support enhances the woman's confidence to seek early treatment for herself [30].

The current study reflected a positive association between the level of education and stage of behavioral adoption of BSE. This indicated that the level of education influences the performance of BSE as women with high education tends to be able to obtain information on breast cancer by themselves. In the process of obtaining information, they became more aware of the benefits of early breast cancer detection [18]. This is in line with previous studies that indicated high education will increase breast self-exploration practice [24],[31]–[33] in detecting any difference in their breast [13] due to adequate knowledge and skills to perform BSE. Further, women with higher income were found to perform BSE more frequently compared to those in the lower income as they usually have a healthier lifestyle and are more aware of their health [18].

### 4.2. Effects of knowledge of breast cancer on the stage of behavioral adoption of BSE

Findings of the study indicated that breast health knowledge is still insufficient amongst the women in Kuantan, Pahang. The lack of knowledge of breast cancer and BSE could be due to insufficient source of knowledge from the media such as newspapers, magazines [28], television [34] as well as from the healthcare providers [35]. This finding is in line with that of a previous study carried out on female Malaysian university students [2]. The role of healthcare providers has been highlighted in many literatures to promote and manage health diseases such as breast cancer [9],[36]. Health promotion programs on BSE such as providing training will increase the regular performance of BSE amongst women [9] as it will increase the women's confidence in performing BSE by themselves and thus will lead to a regular practice of BSE [36]. A previous study indicated that adequate knowledge [18],[19],[22] on breast screening methods induced women to be more aware of any differences in their breasts [37], which encourages them to perform it regularly [17]. This finding is similar to the finding of a previous study that found women with higher knowledge of breast cancer were more likely to perform BSE and have mammograms carried out regularly [27].

In general, women were found to be more likely to be in the relapse stage compared to their current stage when they did not trust their technique in performing BSE [26]. However, women can be at the action stage but still have poor knowledge of breast cancer when they perceived BSE health beliefs wrongly [38] resulting in them not performing BSE correctly [27]. In due course, they are more likely to relapse in performing BSE. Thus, the right and accurate knowledge of breast cancer is very crucial in increasing the awareness of the importance of performing BSE correctly for early breast cancer detection [38].

The findings of this study could help in the creation of interventions tailored to encourage women to progress towards the maintenance stage of BSE behavioral adoption. Further, understanding the contribution of women's socio-demographic characteristics and knowledge on the behavioral adoption of BSE can lead to risk reduction from relapse of behavioral adoption. This is vital for the success of screening programs, clinical care and policy development as well as to design community education programs to detect breast cancer early. The findings of the study may provide a baseline assessment for future intervention programs to promote early detection and management of breast cancer.

## 5. Limitations

This study has several limitations. The study is limited to women in Kuantan, Pahang to elicit the association of socio-demographic characteristics and knowledge of breast cancer on their stage of behavioral adoption of BSE. As such, the data cannot be generalized to Malaysian women. Further, as this study is a quantitative study, aspects such as feelings and actions of the respondents cannot be known to provide depth and detail pertaining to their attitude, feeling and behavior. Additionally, as this study is a quantitative study, it may not have captured the entire range of knowledge of breast cancer, practices and experiences of breast screening due to the invariability of racial dispersion of respondents. Some of the responses may have been biased particularly for those who completed their survey in the presence of researchers. As the questionnaire focuses on breast screening practices as positive behavior, it is possible the respondents gave more socially desirable answers. Lastly, the researcher did not confirm whether respondents knew the correct method to perform BSE even though they indicated that they perform regular BSE.
