# Supplementary material for: The relationship of socio-demographic characteristics and knowledge of breast cancer on stage of behavioral adoption of breast self-examination
Source: AIMS Public Health. 2020 Aug 6;7(3):620–33. doi: 10.3934/publichealth.2020049 (PMC7505780; doi:10.3934/publichealth.2020049)
Supplement: Supplementary file 1 [file publichealth-07-03-049-s001.pdf]

AIMS Public Health

AIMS Public Health, 7(3): 620–633.
DOI: 10.3934/publichealth.2020049
Received: 02 June 2020
Accepted: 31 July 2020
Published: 06 August 2020

http://www.aimspress.com/journal/aimsph

Research article

The relationship of socio-demographic characteristics and knowledge of breast cancer on stage of behavioral adoption of breast self-examination

Soo-Foon Moey*, Aaina Mardhiah Abdul Mutalib, Norfariha Che Mohamed and Nursyahirah Saidin

Department of Diagnostic Imaging & Radiotherapy, Kulliyyah of Allied Health Sciences, International Islamic University Malaysia (IIUM), Kuantan Campus, Pahang, Malaysia

*** Correspondence:** Email: moeysf@iium.edu.my; Tel: +60127751439.

Appendix

Appendix A. Criteria of the respondents.

	Inclusion Criteria Worksheet
✔ if YES	Inclusion Criteria
	1) Female
	2) Malaysian citizen
	3) Aged between 35 and 70 years old
	4) Understand or read and write in Bahasa Malaysia or English
	5) Living in the Kuantan District
	Exclusion Criteria Worksheet
✔ if YES	Exclusion Criteria
	1) Not Malaysian citizen
	2) Aged less than 35 years or more than 70 years
	3) Cannot understand or read in either Bahasa Malaysia or English
	4) Do not live in Kuantan District

Appendix B. Stage of behavioral adoption of BSE.

Stage	Definition
Pre-contemplation	Never done BSE, and do not intend to do it in the next six months<br>- Women at this stage are usually uninformed or underinformed about the consequences of their behavior. Additionally, there are situations in which they tried to make a change but failed multiple times and become demoralized due to their unsuccessful attempt.
Contemplation	Never done BSE, and intend to do it within the next six months<br>- At this stage, they start to recognize a problem in their behavior and begin to have a thought and consideration to change their behavior however, may still feel unsure toward changing their behavior.
Preparation	Never done BSE, and intend to do it within the next one month<br>- Women are ready to act within a short period of time and they start to make small steps towards the change. At this stage, they believe that by changing their behavior, it will lead to a healthier life.
Action	Regularly done and intend to do it over the years<br>- Women have recently changed their behavior and intend to keep moving forward with the stage.
Maintenance	Regularly done more than six months and still going to do it over the years<br>- Women still intend to maintain the behavior and work to maintain their behavior to avoid the relapse stage.
Relapse	Been doing BSE but not going to do it again<br>- Women at this stage return to their unhealthy behavior after a certain time because they feel disappointed and frustrated after failing for a certain course of time.

AIMS Press

© 2020 the Author(s), licensee AIMS Press. This is an open access article distributed under the terms of the Creative Commons Attribution License (http://creativecommons.org/licenses/by/4.0)
